# Association of Malnutrition with Risk of Acute Kidney Injury: A Systematic Review and Meta-Analysis

**DOI:** 10.1155/2023/9910718

**Published:** 2023-09-26

**Authors:** Xiang Xiang, Xinchen Zhu, Lijuan Zhang

**Affiliations:** ^1^Geriatric Diseases Institute of Chengdu/Cancer Prevention and Treatment Institute of Chengdu, Department of Critical Care Medicine, Chengdu Fifth People's Hospital (The Second Clinical Medical College, Affiliated Fifth People's Hospital of Chengdu University of Traditional Chinese Medicine), Chengdu 611137, China; ^2^Department of Internal Medicine, Traditional Chinese Medicine Hospital of Wenjiang District, Chengdu 611130, China

## Abstract

**Background:**

Acute kidney injury (AKI) is a complex clinical syndrome of hospitalization that may be affected by undernutrition and metabolic changes. The aim of this meta-analysis was to systematically assess the association between malnutrition and the risk of prevalent AKI.

**Materials and Methods:**

We searched PubMed, Embase, Ovid MEDLINE, Web of Science, and Chinese databases (WANFANG, VIP, and CKI) from database inception until May 1, 2023, for studies evaluating the association of malnutrition with the risk of AKI. Summary odds ratios (ORs) were estimated using a random-effects model.

**Results:**

We identified 17 observational studies, which included 273,315 individuals. Compared with patients with normal nutritional status, those with malnutrition had a 125% increased risk of prevalent AKI (pooled ORs, 2.25; 95% confidence interval, 1.80–2.82). Malnutrition was also significantly associated with prevalent AKI across all subgroups when subgroup analyses were performed on covariates such as region, study design, age, sample size, malnutrition assessment method, patient characteristics, covariate adjustment degree, and risk of bias. Meta-regression models demonstrated no significant differences in AKI risk between patients with malnutrition and without malnutrition.

**Conclusions:**

Our results suggest that malnutrition may be a potential target for AKI prevention. However, well-designed studies with ethnically or geographically diverse populations are needed to evaluate strategies and interventions to prevent or slow the development and progression of AKI in malnourished individuals.

## 1. Introduction

Acute kidney injury (AKI) is a complex clinical syndrome caused by a range of factors in various clinical settings and is characterized by a sudden but often reversible decline in the estimated glomerular filtration rate (eGFR) and the resultant accumulation of metabolic waste products [1]. Annually, up to 13.3 million people worldwide are affected by AKI, and approximately 1.7 million die of AKI [2]. Although renal replacement therapy is widely used in critically ill patients with AKI, these patients still face an increased risk of mortality and irreversible kidney function deterioration, with rapid progression to chronic kidney disease (CKD) [3]. Therefore, the identification of effective predictive factors for AKI prevention remains critical.

Malnutrition is defined as a physical state of imbalanced nutrition that is significantly associated with increased length of stay, complication rates, healthcare costs, and mortality in hospitalized patients [4, 5]. A recent study that collected data from 238 million emergency department visits reported the prevalence of malnutrition diagnosed by the International Classification of Diseases (ICD), 9th Edition, and diagnosis codes in elderly people increased from 2.5% in 2006 to 3.6% in 2014 [6]. Meanwhile, approximately 15%–80% of hospitalized patients and more than 50% of renal inpatients were reported to be at undernutrition risk [7, 8]. AKI is a common complication of hospitalization and is affected by undernutrition and metabolic changes. Therefore, malnourished patients may be more prone to progression to AKI [9].

Emerging evidence has suggested that malnutrition is a predictor of AKI. A retrospective study of 46,549 Chinese inpatients demonstrated that a malnutrition level ≥3, as assessed using the nutritional risk screening 2002 (NRS-2002), was strongly associated with prevalent AKI [10]. Another retrospective multicenter study of 3,185 patients with acute coronary syndrome found that NRS-2002 ≥3 was associated with an approximately 1.36-fold likelihood of developing AKI [11]. A study of 4,386 patients who underwent coronary angiography showed that moderate malnutrition, evaluated using the Controlling Nutritional Status (CONUT) score and the prognostic nutritional index (PNI), was not associated with an increased risk of AKI [12]. Given the contradictory relationship between malnutrition and the risk of AKI in previous studies, a meta-analysis is imperative to assess the association between malnutrition and the risk of incident AKI. Therefore, the present systematic review and meta-analysis aimed at providing information on the association between malnutrition and the risk of AKI based on current evidence, according to the Preferred Reporting Items for Systematic Reviews and Meta-Analyses (PRISMA) guidelines.

## 2. Methods

### 2.1. Registration of Review Protocol

We reported our meta-analysis review in compliance with the Preferred Reporting Items for Systematic Reviews and Meta-Analyses (PRISMA) guidelines [13] (PRISMA checklist; see Supplementary Appendix S1), and the protocol for this systematic review was registered in INPLASY (registration number: INPLASY202320062).

### 2.2. Data Sources and Search

We searched for potential publications in PubMed, Embase, Ovid MEDLINE, Web of Science, and Chinese databases (WANFANG, VIP, and CKI) from database inception until May 1, 2023. The search terms were “acute kidney injury,” “acute renal injury,” “AKI,” “malnutrition,” “nutritional deficiency,” “nutritional deficiencies,” “undernutrition,” “malnutrition” and “Nutritional Risk Index,” “Geriatric Nutritional Risk Index,” “Subjective Global Assessment,” “Mini Nutritional Assessment-Screening Form,” “Malnutrition Universal Screening Tool,” “Nutritional Risk Screening 2002,” and “Prognostic nutritional index.” The details are shown in the Supplementary Appendix S2.

### 2.3. Study Selection

All articles retrieved using our search strategies were assessed by screening titles and abstracts. Study selection was conducted by two authors independently. In cases of disagreement on the inclusion or exclusion of studies, consensus was reached through discussion with a third author.

### 2.4. Inclusion and Exclusion Criteria

The inclusion criteria were observational studies, studies investigating the association between malnutrition and AKI risk, studies on populations of any sex or ethnicity, and studies with a clear diagnosis of malnutrition and AKI. Animal studies, non-English-language studies, and study types such as review, conference, letter, case report, and comments were excluded.

### 2.5. Data Extraction

Data were independently extracted from the included studies by two authors. Any disagreement was resolved by consensus between the two authors. The following data were extracted from the included studies: year of publication, country, participant characteristics, sample size, malnutrition assessment tools, adjusted variables, and study quality. Disagreements between the two authors were resolved through discussion until consensus was reached.

### 2.6. Quality Assessment

In the evaluation of potential biases within the selected studies, two independent reviewers utilized the Risk of Bias in Nonrandomized Studies of Interventions (ROBINS-I) tool [14]. The ROBINS-I tool, comprised of seven domains, facilitated the assessment of bias arising from factors such as confounding, selection of participants, exposure assessment, misclassification during follow-up, missing data, outcome assessment, and selective reporting. The risk associated with each domain was systematically assessed by the reviewers, who assigned one of the following ratings: low, moderate, serious, critical, or no information. In the event of any discrepancies in ratings, a senior investigator was brought in to arbitrate and resolve the disagreement. Detailed descriptions and decision-making parameters for each of the ROBINS-I tool's domains are comprehensively outlined in Supplementary Appendix S3. This ensures a transparent and replicable process for bias assessment, supporting the robustness of our study's methodology.

### 2.7. Meta-Analysis

For data analysis, we used the “metaprop” function in the “meta” package of R (version 4.1.3). To enable accurate calculation and input of effect sizes and thus ensure the reliability of our results, we used the “metagen” function. In our research, we dealt with dichotomous data, for which we calculated the odds ratios (ORs) and their corresponding 95% confidence intervals (CIs). In addition, to evaluate the heterogeneity among the studies, we utilized the chi-squared test for Cochran's *Q* statistic and *I*^2^. Both of these tools helped us quantify the extent of variability in effect estimates due to heterogeneity rather than chance. We capitalized on both fixed- and random-effects models, depending on the level of heterogeneity. In instances of significant heterogeneity, the preference was given to the random-effects model. More specifically, we employed the Der Simonian and Laird method within the context of the random-effects model. This method considers the variance of the effect sizes across the studies, providing a more accurate computation of weights for meta-analysis. This approach thereby offers a more reliable estimate of the overall effect size, enhancing the credibility of our findings. Moreover, to identify potential sources of heterogeneity, we conducted subgroup and meta-regression analyses. We delineated subgroups based on various factors, including region, malnutrition assessment method, participant demographics, sample size, the degree of covariate adjustment, and study quality. This comprehensive analysis enabled us to delve deeper into the influence of these factors on our results. In addition, to evaluate whether the overall estimate depended on a single study, we conducted a sensitivity analysis by removing any of the included studies. Moreover, we used funnel plots to visualize the publication bias of the study data and further used Egger's test to objectively assess potential publication bias.

## 3. Results

### 3.1. Study Selection

**Figure 1:**
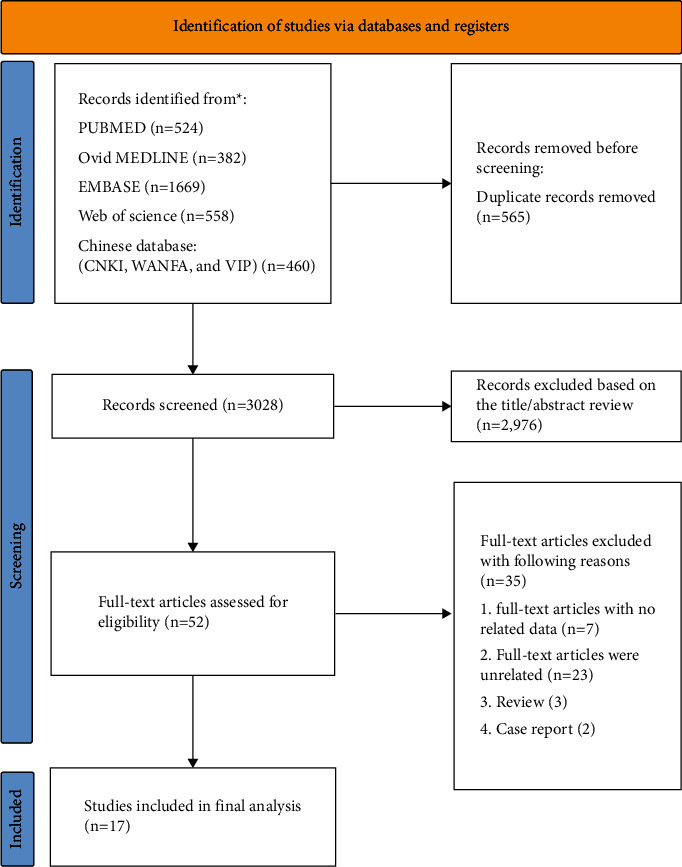
PRISMA flowchart of the meta-analysis.

A flowchart of the literature selection process is presented in Figure 1. Through database searches of PubMed, Embase, Ovid MEDLINE, Web of Science, and Chinese databases (WANFANG, VIP, and CKI), 3593 articles were selected for subsequent filtering. Of these, 565 duplicate studies were excluded. After checking the title and abstract of each paper and excluding inconsistent literature types, 52 studies were found to be pertinent to the research topic. Among them, seven articles were excluded due to inaccessible data, twenty-three articles were excluded after a detailed review of the full text, three were review studies, and two were case reports. Finally, 17 original studies were included in the meta-analysis.

### 3.2. Summary of Studies

The characteristics of the included studies are summarized in Table 1. A total of 273,315 individuals were included in three cross-sectional studies [12, 15, 16], five prospective cohort studies [17–21], and nine retrospective cohort studies [10, 11, 22–28]. The population of the original studies was mainly from China [10–12, 17–21, 23, 25, 28]; three were from Turkey [16, 22, 24, 26, 27], and one was from America [15]. Among the 17 studies, 11 studies included patients undergoing coronary angiography (CAG) or percutaneous coronary angiography [11, 12, 16, 17, 19, 21, 23–26, 28], two included patients undergoing coronary artery bypass [22, 27], one was related to pediatric severe sepsis [15], and three included patients with other diseases [10, 18, 20]. Malnutrition was mainly assessed using the CONUT score, geriatric nutritional risk index, Modified Nutrition Risk in Critically Ill Patients score, PNI, and NRS-2002. AKI was assessed using the Kidney Disease Improving Global Outcomes and contrast-associated AKI (CA-AKI) criteria.

### 3.3. Overall Assessment of Evidence Quality

Utilizing the ROBINS-I instrument for evaluation, it was discerned that four studies presented a moderate overall risk of bias. Eleven studies were identified as having a serious overall risk of bias. In addition, two studies were found to show a serious overall risk of bias. Supplementary Appendix S3 presents the detailed assessment of the risk of bias for each domain.

### 3.4. Association of Malnutrition with Prevalent AKI

**Figure 2:**
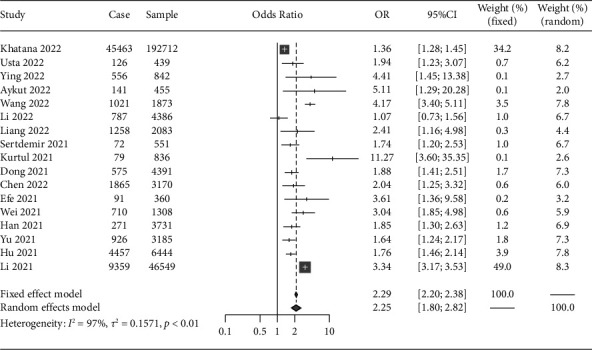
Association between malnutrition and acute kidney injury risk.

Compared with patients with normal nutritional status, those with malnutrition had a 125% increased risk of prevalent AKI (pooled OR, 2.25; 95% CI, 1.80–2.82). Given their combined *I*^2^ = 97.0% and *P* < 0.01 and that the included studies were derived from multiple study settings and their populations, relevant effect values were pooled using a random-effects model (Figure 2).

Owing to the heterogeneity of the aggregated results, it was necessary to explore the sources of heterogeneity. Subgroup analyses were performed on covariates, such as region, study design, age, sample size, malnutrition assessment method, patient characteristics, covariate adjustment degree, and study quality, demonstrating that malnutrition was significantly associated with prevalent AKI across all subgroups. Detailed results are presented in Table 2 and Supplementary Figures 1–8. A univariate meta-regression analysis was then used to examine the potential source of heterogeneity, which revealed no significant differences in AKI risk between patients with and without malnutrition in terms of region (standard error (SE) = 0.29, *P*=0.88), study design (standard error (SE) = 0.44, *P*=0019), sample size (SE = 0.40, *P*=0.20), mean or median patient age (SE = 0.29, *P*=0.60), malnutrition assessment method (SE = 0.29, *P*=0.73), patient characteristics (SE = 0.28, *P*=0.62), adjusted/unadjusted confounders (SE = 0.30, *P*=0.51), or risk of bias (SE = 0.57, *P*=0.47) (Table 3).

### 3.5. Publication Bias and Sensitivity Analysis

To test the publication bias of the included studies, we used funnel plots and Egger 's test, and the results showed that the funnel plots assessing the risk of publication bias showed asymmetry, but Egger' s test result was *t* = 0.16 and *P*=0.87, suggesting that the included studies had no publication bias (Supplementary Figure 9). Sensitivity analyses were also performed to determine the stability and reliability of the results and the extent to which the individual studies influenced the results. The effect size after excluding individual studies was in agreement with the total combined effect size, indicating that our results were robust to a certain extent (Supplementary Figure 10).

## 4. Discussion

To the best of our knowledge, the present meta-analysis of the association between malnutrition and the risk of prevalent AKI is the most comprehensive assessment of this association to date. In this meta-analysis of 17 observational studies, with aggregate data from 273,315 patients from different regions, we found that malnutrition was significantly associated with an approximately 2.25-fold increased risk of prevalent AKI. The results of our meta-analysis indirectly support the concept that interventions against malnutrition may be effective targets for the prevention of AKI.

To date, the possible mechanisms underlying the observed association between malnutrition and an increased risk of AKI are currently unclear, but they can be explained by several factors. Albumin, a popular biomarker of nutritional status in clinically stable conditions, is the most abundant circulating protein and plays an essential role in antioxidant, anti-inflammatory, and antiplatelet aggregation activities [29–31]. Low albumin levels in malnourished patients may contribute to the development of AKI through the deterioration of endothelial function and oxidative inflammatory pathways [32, 33]. Yu et al. reported a higher incidence of AKI in patients with hypoalbuminemia (serum albumin level <3.4 mg/dL) (10.7%) than that in those with normal albumin levels (4.1%) [34], and the results from a meta-analysis of 68,000 participants across a diverse range of settings confirmed that hypoalbuminemia was an independent predictor for the development of AKI [34, 35]. In addition, hypercholesterolemia and low high-density lipoprotein levels, which are indicators of underlying malnutrition, have long been regarded as important risk factors for the development and progression of cardiovascular diseases [36–38], whereas hypocholesterolemia has been reported to be significantly associated with a 1.5-fold risk of 30-day mortality and a 1.3-fold risk of 3-year mortality in patients with coronary artery disease undergoing percutaneous coronary intervention (PCI) [39]. Previous studies have also demonstrated that a low preoperative high-density lipoprotein cholesterol concentration was associated with an increased risk of AKI after cardiac surgery, and low cholesterol levels were significantly associated with worse survival in patients with AKI [40–43].

According to the subgroup analysis, the source of heterogeneity is partly explained by the differences in patient characteristics. Eleven studies explored the association of malnutrition with the risk of CA-AKI among patients undergoing CAG or PCI. CA-AKI is a common phenomenon in patients following contemporary PCI and is characterized by an abrupt decline in eGFR after intravascular administration of iodinated contrast media. Studies reported that CA-AKI had an incidence of 6%–18% in patients undergoing PCI [44–46] and that it was associated with an approximately 2.0-fold increased risk of the 2-year rate of net adverse clinical events [47]. Thus, effective early screening and preventive strategies for those with high-risk CA-AKI are critical. In the present meta-analysis, 11 studies with 24,843 patients were included to explore the role of malnutrition in the risk of CA-AKI; it was found that malnutrition was associated with a 2.09-fold increased risk of CA-AKI. In addition, malnourished patients with CA-AKI had a significantly higher risk of all-cause mortality than patients with malnutrition. A multicenter prospective cohort of 2,083 patients undergoing PCI demonstrated that malnourished patients with CA-AKI had a higher risk of all-cause mortality than did those without [19]. Therefore, clinicians should assess and monitor the nutritional status of patients undergoing PCI. Malnutrition was also associated with a high risk of AKI in patients undergoing abdominal surgery. Sim et al. studied 3,543 patients who underwent colorectal cancer surgery and found that a high preoperative PNI was significantly associated with a low risk of postoperative AKI (OR, 0.96; 95% CI, 0.93–0.99, *P*=0.003) [48]. Another study of 817 patients who underwent hepatectomy for hepatocellular carcinoma demonstrated that a high preoperative PNI was significantly associated with a lower prevalence of postoperative AKI (OR, 0.92; 95% CI, 0.85–0.99; *P*=0.021) [49]. Thus, more attention should be paid to preoperative nutritional management, which can provide useful information about postoperative AKI and prognosis in patients undergoing abdominal surgery.

Another important source of heterogeneity can be partly explained by the differences in malnutrition assessment methods. To date, there is a lack of unified and standard measurement tools for malnutrition; thus, the prevalence of malnutrition varies when different measurement tools are used. Fortunately, the results from the subgroup analysis showed that malnutrition was associated with an increased risk of AKI across all groups of assessment tools for malnutrition. The NRS-2002 score, developed by the European Society for Clinical Nutrition and Metabolism, has been widely used in clinical malnutrition screening in various diseases, including CKD. The parameters of the NRS-2002 score, such as decreased body mass index and intensive care admission, were also significantly associated with the occurrence of AKI [10, 50]. In our study, malnutrition assessed by NRS-2002 was associated with a 2.38-fold risk of AKI, and it showed a robust ability to predict AKI, with areas under the curve of 0.67 for the univariate model and 0.78 for the multivariate model [10]. The CONUT score, which is calculated using the serum albumin level and lymphocytes, is an efficient and simple tool for detecting malnutrition. Pooled analysis demonstrated that malnutrition assessed using the CONUT score was associated with a 2.37-fold risk of AKI. In addition, Wei et al. found that, compared with the mild malnutrition group, the moderate-to-severe malnutrition group showed a higher risk of CA-AKI [28]. The PNI, which is calculated using the serum albumin level and total lymphocyte count, is another tool for assessing nutrition and reflects chronic inflammation and immunity [51]. Malnutrition assessed by the PNI was associated with a 1.79-fold increased risk of AKI. Yu et al. demonstrated that each decrease in PNI score was associated with a 1.8% increased risk of AKI [34].

In our meta-analysis, the source of heterogeneity was investigated through regression analysis. Intriguingly, our findings indicate that study design could potentially be a notable source of heterogeneity. Specifically, there was a significant difference between cohort studies and cross-sectional studies (SE = 0.44, *P*=0.019). This could be attributable to the fundamental differences in methodology between these two types of study design. In cohort studies, participants are followed over time, and data about them is collected at regular intervals, allowing for the observation of changes and the development of outcomes over time. In contrast, cross-sectional studies provide a snapshot of a population at a specific point in time, which could lead to different findings and interpretations [52, 53]. This discrepancy in design could introduce variability, or heterogeneity, into our meta-analysis. Consequently, future meta-analyses should consider the potential impact of study design and address this source of heterogeneity to ensure the robustness and reliability of their findings.

Our meta-analysis has some limitations that are strictly inherent to the design of the eligible studies. First, our meta-analysis could not prove causality between malnutrition and the risk of AKI because of the observational design of the included studies. Second, heterogeneity was high in the pooled analyses, and the meta-regression analysis failed to define the source of heterogeneity, which may have affected the reliability of our results. Third, the included studies were mostly conducted in China and Turkey, and there was a significant publication bias in the funnel plot, which may have weakened our ability to evaluate the strength of the association between malnutrition and the risk of prevalent AKI in other countries. Fortunately, the significant association between malnutrition and the risk of prevalent AKI has also been confirmed in several studies conducted in America and Korea [15, 48, 49]. Nevertheless, further well-designed cohort studies with ethnically or geographically diverse populations are required to confirm whether this further amplifies the increased risk of developing AKI in malnourished patients in other regions. Fourth, we excluded studies in languages other than English and Chinese, which may introduce language bias. Despite these limitations, the present meta-analysis has several strengths. To our knowledge, this is the first study to examine the correlation between malnutrition and the risk of acute kidney injury (AKI) from a global perspective. Although previous studies have deepened our understanding of the relationship between malnutrition and contrast-induced nephropathy [54], it is crucial to note that contrast-induced nephropathy and AKI are two different pathologies with distinct etiologies, pathophysiological mechanisms, and clinical presentations. In our research, we focus on the association between malnutrition and AKI. This field has not been extensively discussed in prior research and is often confounded by other variables, leading to significant controversy and ambiguity over the correlation between malnutrition and AKI. Our study capitalizes on data from diverse countries and populations, enabling a more accurate and universal reflection of malnourished individuals, thus providing higher value for routine clinical practice; furthermore, the included subjects are likely to be an accurate reflection of individuals with malnutrition seen in routine clinical practice.

In conclusion, the results of the present meta-analysis provide evidence that malnutrition is significantly associated with an increased AKI prevalence. Our findings suggest that malnutrition may be a potential target for AKI prevention. However, well-designed studies with ethnically or geographically diverse populations are needed to evaluate strategies and interventions to prevent or slow the development and progression of AKI in malnourished individuals.

## Figures and Tables

**Table 1:** Main characteristics of included studies.

First author, years	Region	Male (%)	Age (years)	Source of participants	Malnutrition assessment method	AKI	Study quality
Khatana 2022 [15]	America	50.60	1–20	192,712 hospitalizations due to pediatric severe sepsis	Unclear	ICD 9 codes and ICD 10 codes	10

Usta 2022 [27]	Turkey	77.70	>65	439 patients who underwent cardiac surgery with cardiopulmonary bypass	Controlling Nutritional Status score	Stage 1: an increase ≥0.3 mg/dL in creatinine from baseline or an increase of 1.5–1.9 times the initial value	8
						Stage 2: an increase of 2.0–2.9 times the basal creatinine value	
						Stage 3: more than 3-fold increase from baseline or ≥4.0-mg/dL increase in Scr or need for renal replacement therapy	

Ying 2022 [21]	China	69.60	61.80 ± 11.27	842 patients who were diagnosed with chronic heart failure following CAG or PCI	Controlling Nutritional Status score	An increase in the Scr level >0.3 mg/dL or >50% from the baseline level within the first 48 h after the procedure	9

Aykut 2022 [22]	Turkey	82.20	>70	455 patients who underwent on-pump coronary artery bypass grafting	Geriatric nutritional risk index	The maximum change in Scr from the preoperative baseline levels	7

Wang 2022 [20]	China	60.40	74 (66–81)	1,873 elderly patients in the intensive care unit	The Modified Nutrition Risk in Critically Ill (mNUTRIC) score	Increase in Scr by ≥0.3 mg/dL (≥26 mmol/L) within 48 h or ≥1.5 times baseline, which is known or presumed to have occurred within the prior 7 days or urine volume of 0.5 mL/kg/h for 6 h	9

Li 2022 [12]	China	66.00	67.1 ± 10.8	4,386 patients undergoing CAG	Prognostic nutritional index	An increase in Scr by more than 44 *μ*mol/L (0.5 mg/dL) or 25% within 72 h of intravascular contrast injection	9

Liang 2022 [19]	China	79.00	62.8 ± 11.1	2,083 patients undergoing PCI	Controlling nutritional status score	An increase in scr >0.3 mg/dL or >50% than the baseline level within 48 h after the intervention	9

Sertdemir 2021 [16]	Turkey	64.00	62.5 ± 10.7	51 patients with ACS admitted to the coronary angiography laboratory for emergency PCI	Prognostic nutritional index	An increase in Scr ≥0.3 mg/dL or ≥50% from the baseline Scr levels within 48 h after PCI	7

Kurtul 2021 [26]	Turkey	76.00	58 ± 12	836 patients with STEMI treated with primary PCI	Prognostic nutritional index	An increase in plasma creatinine level ≥0.5 mg/dL or ≥25% increase from the baseline level within 3 days after exposure to contrast material due to primary PCI	8

Dong 2021 [23]	China	73.38	68.71 ± 9.95	4,391 patients with CKD and CAD	Prognostic nutritional index	An increase in Scr of 0.5 mg/dL or 25% within the first 48 h after the procedure	9

Chen 2022 [17]	China	76.69	63.1 ± 10.7	3,170 CAD patients	Controlling Nutritional Status score	An increase in Scr level >0.3 mg/dL or >50% from the baseline level within the first 48 h after the procedure	9

Efe 2021 [24]	Turkey	63.30	69 (67–72)	360 consecutive patients with CAG	Geriatric nutritional risk index	An increase of 0.5 mg/dL in plasma creatinine levels or a 25% increase in basal creatinine within 72 h after the procedure	7

Wei 2021 [28]	China	72.10	>75	1,308 patients aged >75 years undergoing PCI	Controlling Nutritional Status score	An absolute increase ≥0.3 mg/dL or ≥50% from the baseline Scr levels within 48 h after exposure to contrast media	8

Han 2021 [25]	Korea	71.40	65.4 ± 11.3	3,731 patients who received PCI	Prognostic nutritional index	An increase in Scr ≥0.3 mg/dL or 1.5-fold than the baseline value within 48 h after PCI	9

Yu 2021 [34]	China	74.80	65.9 ± 13.08	3,185 ACS patients	Nutrition Risk Screening 2002	AKI stage 1: Scr concentration >26.5 mmol/L (0.3 mg/dL) within 48 h, a 1.5-1.9-fold increase in Scr from the baseline value, or urine output <0.5 mL/kg/h for 6–12 h	9
						AKI stage 2: a 2.0-2.9-fold increase in Scr from the baseline value or urine output <0.5 mL/kg/h for 12 h	
						AKI stage 3: scr concentration >353.6 mmol/L (4.0 mg/dL), a >3.0-fold increase in Scr from the baseline value, urine output <0.3 mL/kg/h for 24 h, or anuria for 12 h	

Hu 2021 [18]	China	40.50	67.1 ± 14.9	6,444 patients from the Medical Information Mart for Intensive Care	Prognostic nutritional index	Scr criteria within 7 days after hospital admission	8

Li 2021 [10]	China	56.70	57.6 ± 14.8	46,549 inpatients	Nutrition Risk Screening 2002	Scr level >26.5 mmol/L within 48 h, an increase in Scr to >1.5-fold the baseline-confirmed value or an increase presumed to have occurred within 7 d, or (3) urine output <0.5 mL/kg/h for >6 h	9

ACS, acute coronary syndrome; AKI, acute kidney injury; CAD, coronary artery disease; CAG, coronary angiography; CKD, chronic kidney disease; ICD, International Classification of Diseases; PCI, percutaneous coronary angiography; Scr, serum creatinine; STEMI, ST-elevation myocardial infarction.

**Table 2:** Subgroup analyses examining the associations between malnutrition and AKI risk.

Category	Participants (*n*)	OR	95% CI	*I* ^2^ (%)	*P* (*z*-text)	Effect model
Total	273,315 (*n* = 17)	2.25	(1.80–2.82)	97.0	<0.01	Random
*Region*						
America	192,712 (*n* = 1)	1.36	(1.28–1.45)	N/A	<0.01	Random
East Asia	77,962 (*n* = 11)	2.23	(1.73–2.89)	92.0	<0.01	Random
West Asia	2,641 (*n* = 5)	3.14	(1.64–6.02)	67.0	<0.01	Random
*Study design*						
Cohort study	75,666 (*n* = 14)	2.55	(2.04–3.20)	87.0	<0.01	Random
Cross-sectional study	197,649 (*n* = 3)	1.36	(1.28–1.45)	38.0	<0.01	Fixed
*Sample number ≥1000*						
Yes	269,832 (*n* = 11)	2.07	(1.61–2.66)	98.0	<0.01	Random
No	3,483 (*n* = 6)	3.22	(1.83–5.67)	63.0	<0.01	Random
*The mean/median of age ≥65 years*						
Yes	26,572 (*n* = 10)	2.14	(1.62–2.82)	87.0	<0.01	Random
No	246,743 (*n* = 7)	2.52	(1.65–3.84)	99.0	<0.01	Random
*Malnutrition assessment method*						
Controlling Nutritional Status score	7,842 (*n* = 5)	2.37	(1.84–3.04)	0.0	<0.01	Fixed
Geriatric nutritional risk index	815 (*n* = 2)	4.05	(1.83–8.99)	0.0	<0.01	Fixed
Nutrition Risk Screening 2002	49,734 (*n* = 2)	2.38	(1.19–4.76)	96.0	0.015	Random
Prognostic nutritional index	20,339 (*n* = 6)	1.79	(1.38–2.31)	71.0	<0.01	Random
Others	19,4585 (*n* = 2)	2.37	(0.79–7.10)	99.0	0.122	Random
*Patient characteristics*						
Patients undergoing coronary artery bypass	894 (*n* = 2)	2.14	(1.38–3.30)	41.0	0.031	Fixed
Patients undergoing CAG or PCI	24,843 (*n* = 11)	2.09	(1.63–2.69)	63.0	<0.01	Random
Other inpatients	247,578 (*n* = 4)	2.40	(1.43–4.02)	99.0	<0.01	Random
*Covariate adjustment degree*						
Adjusted OR	30,322 (*n* = 12)	1.94	(1.61–2.34)	57.0	<0.01	Random
Unadjusted OR	242,993 (*n* = 5)	2.51	(1.63–3.85)	99.0	<0.01	Random
*Risk of bias*						
Low	10,099 (*n* = 4)	2.02	(1.60–2.55)	0.0	<0.01	Fixed
Moderate	261,829 (*n* = 11)	2.18	(1.65–2.89)	98.0	<0.01	Random
Serious	1387 (*n* = 2)	4.08	(0.66-25.32)	89.0	0.131	Random

AKI, acute kidney injury; CAG, coronary angiography; PCI, percutaneous coronary angiography; OR, odds ratio; CI, confidence interval.

**Table 3:** Results of the univariate meta-regression analyses examining possible sources of between-study heterogeneity.

Variable	Β (95% CI)	SE	*P*
Region: other vs. East Asia	1.05 (0.58–1.87)	0.29	0.88
Study design: cohort study vs. cross-sectional study	1.86 (1.12–3.08)	0.44	0.019
Sample number ≥1,000: no vs. yes	1.46 (0.81–2.63)	0.40	0.20
Mean/median age ≥65 years: no vs. yes	1.15 (0.67–1.97)	0.29	0.60
Diagnosis of malnutrition: others vs. prognostic nutritional index	1.10 (0.62–1.94)	0.29	0.73
Patient characteristics: other inpatients vs. patients undergoing CAG or PCI	1.12 (0.65–1.91)	0.28	0.62
Covariate adjustment degree: unadjusted vs. adjusted OR	1.18 (0.69–2.03)	0.30	0.51
Risk of bias: moderate vs. other	1.36 (0.56–3.33)	0.57	0.47

CAG, coronary angiography; PCI, percutaneous coronary angiography; OR, odds ratio; CI, confidence interval; SE, standard error.

## Data Availability

The original contributions presented in this study are included in the article/Supplementary Materials; further inquiries can be directed to the corresponding author.

## Supplementary Materials

Supplementary Materials Supplementary Figure 1: subgroup analysis for malnutrition-related risk of prevalent acute kidney injury by region. Supplementary Figure 2: subgroup analysis for malnutrition-related risk of prevalent acute kidney injury by sample number. Supplementary Figure 3: subgroup analysis for malnutrition-related risk of prevalent acute kidney injury by age. Supplementary Figure 4: subgroup analysis for malnutrition-related risk of prevalent acute kidney injury by malnutrition assessment method. Supplementary Figure 5: subgroup analysis for malnutrition-related risk of prevalent acute kidney injury by patient characteristics. Supplementary Figure 6: subgroup analysis for malnutrition-related risk of prevalent acute kidney injury by covariate adjustment degree. Supplementary Figure 7: subgroup analysis for malnutrition-related risk of prevalent acute kidney injury by study quality. Supplementary Figure 8: sensitivity analysis for the risk of prevalent acute kidney injury in patients with malnutrition. Supplementary Figure 9: funnel plot for the risk of prevalent AKI in malnutrition patient. Supplementary Figure 10: sensitivity analysis for the risk of prevalent AKI in malnutrition patient. Supplementary Table 1: sensitivity analysis for the risk of prevalent AKI in malnutrition patient.
