# Supplementary material for: Association of Malnutrition with Risk of Acute Kidney Injury: A Systematic Review and Meta-Analysis
Source: Int J Clin Pract. 2023 Sep 26;2023:9910718. doi: 10.1155/2023/9910718 (PMC10547578; doi:10.1155/2023/9910718)
Supplement: Supplementary Materials — Supplementary Figure 1: subgroup analysis for malnutrition-related risk of prevalent acute kidney injury by region. Supplementary Figure 2: subgroup analysis for malnutrition-related risk of prevalent acute kidney injury by sample number. Supplementary Figure 3: subgroup analysis for malnutrition-related risk of prevalent acute kidney injury by age. Supplementary Figure 4: subgroup analysis for malnutrition-related risk of prevalent acute kidney injury by malnutrition assessment method. Supplementary Figure 5: subgroup analysis for malnutrition-related risk of prevalent acute kidney injury by patient characteristics. Supplementary Figure 6: subgroup analysis for malnutrition-related risk of prevalent acute kidney injury by covariate adjustment degree. Supplementary Figure 7: subgroup analysis for malnutrition-related risk of prevalent acute kidney injury by study quality. Supplementary Figure 8: sensitivity analysis for the risk of prevalent acute kidney injury in patients with malnutrition. Supplementary Figure 9: funnel plot for the risk of prevalent AKI in malnutrition patient. Supplementary Figure 10: sensitivity analysis for the risk of prevalent AKI in malnutrition patient. Supplementary Table 1: sensitivity analysis for the risk of prevalent AKI in malnutrition patient. [file 9910718.f1.zip › Supplementary appendix S2.Search strategy.docx]

**Search strategy**

**PubMed**

**#1** Acute Kidney Injury[MeSH Terms] **55,531**

**#2** ((((((((((((((Acute Kidney Injur*[Title/Abstract]) OR (Kidney Injury, Acute[Title/Abstract])) OR (Acute Renal Injur*[Title/Abstract])) OR (Renal Injury, Acute[Title/Abstract])) OR (Acute Renal Insufficienc*[Title/Abstract])) OR (Renal Insufficiencies, Acute[Title/Abstract])) OR (Acute Kidney Insufficienc*[Title/Abstract])) OR (Kidney Insufficiency, Acute[Title/Abstract])) OR (Kidney Insufficiencies, Acute[Title/Abstract])) OR (Acute Kidney Failure*[Title/Abstract])) OR (Kidney Failure, Acute[Title/Abstract])) OR (Acute Renal Failure*[Title/Abstract])) OR (Renal Failure, Acute[Title/Abstract])) OR (Acute Renal Failure[Title/Abstract])) OR (AKI[Title/Abstract]) **79,272**

**#3** Malnutrition[MeSH Terms] **134,602**

**#4** ((((((((((Nutritional Deficiency[Title/Abstract]) OR (Nutritional Deficiencies[Title/Abstract])) OR (Undernutrition[Title/Abstract])) OR (Malnourishment[Title/Abstract])) OR (Nutritional Risk Index[Title/Abstract])) OR (Geriatric Nutritional Risk Index[Title/Abstract])) OR (Subjective Global Assessment[Title/Abstract])) OR (Mini Nutritional Assessment – Screening Form[Title/Abstract])) OR (Malnutrition Universal Screening Tool[Title/Abstract])) OR (Nutritional Risk Screening 2002[Title/Abstract])) OR (Prognostic nutritional index[Title/Abstract]) **21419**

**#5** #1 or #2 **95,016**

**#6**  #3 or #4 **149,528**

**#7** #5 and #6 **[524](https://pubmed.ncbi.nlm.nih.gov/?term=%235+and+%236&sort=date&size=200)**

**Embase**

**#1** 'acute kidney injury'/exp OR 'acute kicney injur*':ab,ti OR 'kidney injury, acute':ab,ti OR 'acute renal injur*':ab,ti OR 'renal injury, acute':ab,ti OR 'acute renal insufficienc*':ab,ti OR 'renal insufficiencies, acute':ab,ti OR 'acute kidney insufficienc*':ab,ti OR 'kidney insuficiency, acute':ab,ti OR 'kidney insuficiencies, acute':ab,ti OR 'acute kidney failure':ab,ti OR 'kidney failure acute':ab,ti OR 'renal failure acute':ab,ti OR 'acute renal failure':ab,ti OR 'aki':ab,ti **145248**

**#2** 'malnutrition'/exp OR 'malnutrition' OR 'nutritional deficiency':ab,ti OR 'nutritional deficiencies':ab,ti OR undernutrition:ab,ti OR malnourishment:ab,ti OR 'nutritional risk index':ab,ti OR 'geriatric nutritional risk index':ab,ti OR 'subjective global assessment':ab,ti OR 'mini nutritional assessment – screening form':ab,ti OR 'malnutrition universal screening tool':ab,ti OR 'nutritional risk screening 2002':ab,ti OR 'prognostic nutritional index':ab,ti **240762**

**#7** **#**1and **#**2 **1669**

**MEDLINE**

**#1** exp Acute Kidney Injury/ 5**6175**

**#2** exp Malnutrition/ **135548**

**#3** (Acute Kidney Injur* or Kidney Injury, Acute or Acute Renal Injur* or Renal Injury, Acute or Acute Renal Insufficienc* or Renal Insufficiencies, Acute or Acute Kidney Insufficienc* or Kidney Insufficiency, Acute or Kidney Insufficiencies, Acute or Acute Kidney Failure* or Kidney Failure, Acute or Acute Renal Failure* or Renal Failure, Acute or Acute Renal Failure or AKI).ab,ti. **64093**

**#4** (Nutritional Deficiency or Nutritional Deficiencies or Undernutrition or Malnourishment or nutritional risk index or geriatric nutritional risk index or subjective global assessment or mini nutritional assessment-screening form or malnutrition universal screening tool or nutritional risk screening 2002 or prognostic nutritional index).ab,ti. **20288**

**#5** **#**1 or **#**3 **83191**

**#6** **#**2 or **#**4 **148728**

**#7** **#**5 and **#**6 **382**

**Web of science**

**#1** Acute Kidney Injury (Topic) **56387**

**#2**Acute Kidney Injur* or Kidney Injury, Acute or Acute Renal Injur* or Renal Injury, Acute or Acute Renal Insufficienc* or Renal Insufficiencies, Acute or Acute Kidney Insufficienc* or Kidney Insufficiency, Acute or Kidney Insufficiencies, Acute or Acute Kidney Failure* or Kidney Failure, Acute or Acute Renal Failure* or Renal Failure, Acute or Acute Renal Failure or AKI (Topic) **102883**

**#3** Malnutrition (Topic) **54862**

**#4**Nutritional Deficiency or Nutritional Deficiencies or Undernutrition or Malnourishment or nutritional risk index or geriatric nutritional risk index or subjective global assessment or mini nutritional assessment-screening form or malnutrition universal screening tool or nutritional risk screening 2002 or prognostic nutritional index (Topic) **56658**

**#5** #1 OR #2 **102883**

**#6** #3 OR #4 **97996**

**#7** #6 AND #5 **558**

**CNKI**

#1（摘要：急性肾损伤（精确））AND（摘要：营养（精确）） 208

**WANFANG**

#1摘要:(营养) and 摘要:(急性肾损伤) 精确 237

**VIP**

#1摘要=营养) and 摘要=急性肾损伤 精确 184
