# Supplementary material for: Association of Malnutrition with Risk of Acute Kidney Injury: A Systematic Review and Meta-Analysis
Source: Int J Clin Pract. 2023 Sep 26;2023:9910718. doi: 10.1155/2023/9910718 (PMC10547578; doi:10.1155/2023/9910718)
Supplement: Supplementary Materials — Supplementary Figure 1: subgroup analysis for malnutrition-related risk of prevalent acute kidney injury by region. Supplementary Figure 2: subgroup analysis for malnutrition-related risk of prevalent acute kidney injury by sample number. Supplementary Figure 3: subgroup analysis for malnutrition-related risk of prevalent acute kidney injury by age. Supplementary Figure 4: subgroup analysis for malnutrition-related risk of prevalent acute kidney injury by malnutrition assessment method. Supplementary Figure 5: subgroup analysis for malnutrition-related risk of prevalent acute kidney injury by patient characteristics. Supplementary Figure 6: subgroup analysis for malnutrition-related risk of prevalent acute kidney injury by covariate adjustment degree. Supplementary Figure 7: subgroup analysis for malnutrition-related risk of prevalent acute kidney injury by study quality. Supplementary Figure 8: sensitivity analysis for the risk of prevalent acute kidney injury in patients with malnutrition. Supplementary Figure 9: funnel plot for the risk of prevalent AKI in malnutrition patient. Supplementary Figure 10: sensitivity analysis for the risk of prevalent AKI in malnutrition patient. Supplementary Table 1: sensitivity analysis for the risk of prevalent AKI in malnutrition patient. [file 9910718.f1.zip › Supplementary appendix S3.ROBINS-I.docx]

**Supplementary appendix S3**. Risk-of-bias summary for the studies included in the meta-analysis, using Cochrane risk-of-bias tool ROBINS-I

| **Study** | **Bias due to**  **confounding** | **Bias due to**  **selection of**  **participants** | **Bias due to**  **exposure**  **assessment** | **Bias due to**  **misclassification during**  **folLow-up** | **Bias due to**  **missing data** | **Bias due to**  **measurement of the**  **outcome** | **Bias due to**  **selective reporting**  **of the results** | **Overall**  **judgement** |
| --- | --- | --- | --- | --- | --- | --- | --- | --- |
| Khatana 2022 | Serious | Low | Moderate | Serious | No information | Moderate | Moderate | Moderate |
| Usta 2022 | Moderate | Moderate | Low | Low | No information | Moderate | Moderate | Moderate |
| Ying 2022 | Moderate | Low | Moderate | Low | No information | Moderate | No information | Moderate |
| Aykut 2022 | Moderate） | Moderate | Low | Low | No information | Low | No information | Low |
| Wang 2022 | Serious | Moderate | Low | Moderate | No information | Low | Moderate | Moderate |
| Li 2022 | Low | Moderate | Low | Serious | No information | Moderate | No information | Moderate |
| Liang 2022 | Moderate | Moderate | Low | Low | No information | Low | No information | Low |
| Sertdemir 2021 | Serious | Serious | Moderate | Serious | Moderate | Moderate | No information | Serious |
| Kurtul 2021 | Serious | Serious | Moderate | Moderate | No information | Moderate | No information | Serious |
| Dong 2021 | Moderate | Low | Low | Low | Moderate | Low | No information | Low |
| Chen 2022 | Moderate | Low | Low | Low | Moderate | Low | Moderate | Low |
| Efe 2021 | Moderate | Moderate | Moderate | Moderate | Low | Low | Moderate | Moderate |
| Wei 2021 | Serious | Low | Low | Low | Low | Moderate | Moderate | Moderate |
| Han 2021 | Moderate | Moderate | Moderate | Low | Moderate | Moderate | No information | Moderate |
| Yu 2021 | Moderate | Moderate | Low | Low | Serious | Moderate | Moderate | Moderate |
| Hu 2021 | Moderate | Low | Low | Moderate | Low | Moderate | No information | Moderate |
| Li 2021 | Serious | Moderate | Low | Low | Moderate | Low | Moderate | Moderate |

*Downgraded by one level if >25% of participants in this comparison were from studies at high risk of bias.

†Downgraded by one level if heterogeneity (I^2^) >50%.
