# Supplementary material for: Association of Malnutrition with Risk of Acute Kidney Injury: A Systematic Review and Meta-Analysis
Source: Int J Clin Pract. 2023 Sep 26;2023:9910718. doi: 10.1155/2023/9910718 (PMC10547578; doi:10.1155/2023/9910718)
Supplement: Supplementary Materials — Supplementary Figure 1: subgroup analysis for malnutrition-related risk of prevalent acute kidney injury by region. Supplementary Figure 2: subgroup analysis for malnutrition-related risk of prevalent acute kidney injury by sample number. Supplementary Figure 3: subgroup analysis for malnutrition-related risk of prevalent acute kidney injury by age. Supplementary Figure 4: subgroup analysis for malnutrition-related risk of prevalent acute kidney injury by malnutrition assessment method. Supplementary Figure 5: subgroup analysis for malnutrition-related risk of prevalent acute kidney injury by patient characteristics. Supplementary Figure 6: subgroup analysis for malnutrition-related risk of prevalent acute kidney injury by covariate adjustment degree. Supplementary Figure 7: subgroup analysis for malnutrition-related risk of prevalent acute kidney injury by study quality. Supplementary Figure 8: sensitivity analysis for the risk of prevalent acute kidney injury in patients with malnutrition. Supplementary Figure 9: funnel plot for the risk of prevalent AKI in malnutrition patient. Supplementary Figure 10: sensitivity analysis for the risk of prevalent AKI in malnutrition patient. Supplementary Table 1: sensitivity analysis for the risk of prevalent AKI in malnutrition patient. [file 9910718.f1.zip › Supplementary figure 10. Sensitivity analysis for risk of prevalent AKI in malnutrition patient .pdf]

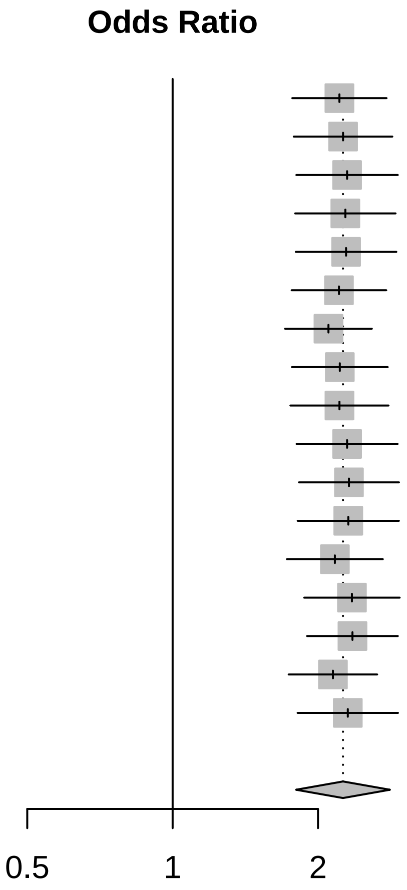

Study	OR	95%−CI	P−value	Tau2	Tau	I^2
Omitting Aykut 2022	2.21	[1.77; 2.77]	< 0.01	0.1550	0.3937	97%
Omitting Liang 2022	2.25	[1.78; 2.85]	< 0.01	0.1678	0.4096	97%
Omitting Dong 2021	2.30	[1.80; 2.93]	< 0.01	0.1738	0.4169	97%
Omitting Chen 2022	2.28	[1.79; 2.89]	< 0.01	0.1724	0.4153	97%
Omitting Usta 2022	2.29	[1.80; 2.91]	< 0.01	0.1724	0.4152	97%
Omitting Ying 2022	2.21	[1.76; 2.77]	< 0.01	0.1551	0.3939	97%
Omitting Wang 2022	2.10	[1.71; 2.59]	< 0.01	0.1155	0.3398	97%
Omitting Efe 2021	2.22	[1.77; 2.79]	< 0.01	0.1586	0.3982	97%
Omitting Wei 2021	2.22	[1.75; 2.80]	< 0.01	0.1629	0.4036	97%
Omitting Han 2021	2.30	[1.81; 2.92]	< 0.01	0.1727	0.4156	97%
Omitting Yu 2021	2.32	[1.83; 2.94]	< 0.01	0.1679	0.4097	97%
Omitting Hu 2021	2.31	[1.82; 2.94]	< 0.01	0.1719	0.4146	97%
Omitting Li 2021	2.17	[1.72; 2.72]	< 0.01	0.1486	0.3855	90%
Omitting Khatana 2022	2.35	[1.87; 2.95]	< 0.01	0.1472	0.3836	89%
Omitting Li 2022	2.36	[1.90; 2.93]	< 0.01	0.1321	0.3634	97%
Omitting Kurtul 2021	2.15	[1.74; 2.65]	< 0.01	0.1313	0.3623	97%
Omitting Sertdemir 2021	2.31	[1.81; 2.93]	< 0.01	0.1705	0.4129	97%
**Random effects model**	**2.25**	**[1.80; 2.82]**	**< 0.01**	**0.1571**	**0.3964**	**97%**

Supplementary figure 10. Sensitivity analysis for risk of prevalent AKI in malnutrition patient
