# Supplementary material for: Association of Malnutrition with Risk of Acute Kidney Injury: A Systematic Review and Meta-Analysis
Source: Int J Clin Pract. 2023 Sep 26;2023:9910718. doi: 10.1155/2023/9910718 (PMC10547578; doi:10.1155/2023/9910718)
Supplement: Supplementary Materials — Supplementary Figure 1: subgroup analysis for malnutrition-related risk of prevalent acute kidney injury by region. Supplementary Figure 2: subgroup analysis for malnutrition-related risk of prevalent acute kidney injury by sample number. Supplementary Figure 3: subgroup analysis for malnutrition-related risk of prevalent acute kidney injury by age. Supplementary Figure 4: subgroup analysis for malnutrition-related risk of prevalent acute kidney injury by malnutrition assessment method. Supplementary Figure 5: subgroup analysis for malnutrition-related risk of prevalent acute kidney injury by patient characteristics. Supplementary Figure 6: subgroup analysis for malnutrition-related risk of prevalent acute kidney injury by covariate adjustment degree. Supplementary Figure 7: subgroup analysis for malnutrition-related risk of prevalent acute kidney injury by study quality. Supplementary Figure 8: sensitivity analysis for the risk of prevalent acute kidney injury in patients with malnutrition. Supplementary Figure 9: funnel plot for the risk of prevalent AKI in malnutrition patient. Supplementary Figure 10: sensitivity analysis for the risk of prevalent AKI in malnutrition patient. Supplementary Table 1: sensitivity analysis for the risk of prevalent AKI in malnutrition patient. [file 9910718.f1.zip › Supplementary figure 3. Subgroup analysis for malnutition-related risk of prevalent AKI by sample number.pdf]

Study	Case	Sample	OR	95%CI	Weight (fixed)	Weight (random)
**Sample number more than 1000: Yes**						
Khatana 2022	45,463	192712	1.36	[1.28; 1.45]	34.2%	8.2%
Wang 2022	1021	1873	4.17	[3.40; 5.11]	3.5%	7.8%
Li 2022	787	4386	1.07	[0.73; 1.56]	1.0%	6.7%
Liang 2022	1258	2083	2.41	[1.16; 4.98]	0.3%	4.4%
Dong 2021	575	4391	1.88	[1.41; 2.51]	1.7%	7.3%
Chen 2022	1865	3170	2.04	[1.25; 3.32]	0.6%	6.0%
Wei 2021	710	1308	3.04	[1.85; 4.98]	0.6%	5.9%
Han 2021	271	3731	1.85	[1.30; 2.63]	1.2%	6.9%
Yu 2021	926	3185	1.64	[1.24; 2.17]	1.8%	7.3%
Hu 2021	4457	6444	1.76	[1.46; 2.14]	3.9%	7.8%
Li 2021	9359	46549	3.34	[3.17; 3.53]	49.0%	8.3%
**Fixed effect model**			**2.29**	**[2.20; 2.38]**	**97.8%**	—
**Random effects model**			**2.07**	**[1.61; 2.66]**	—	**76.6%**
Heterogeneity: $I^2 = 98\%$, $\tau^2 = 0.1523$, $p < 0.01$						
**Sample number more than 1000: No**						
Usta 2022	126	439	1.94	[1.23; 3.07]	0.7%	6.2%
Ying 2022	556	842	4.41	[1.45; 13.38]	0.1%	2.7%
Aykut 2022	141	455	5.11	[1.29; 20.28]	0.1%	2.0%
Sertdemir 2021	72	551	1.74	[1.20; 2.53]	1.0%	6.7%
Kurtul 2021	79	836	11.27	[3.60; 35.35]	0.1%	2.6%
Efe 2021	91	360	3.61	[1.36; 9.58]	0.2%	3.2%
**Fixed effect model**			**2.28**	**[1.76; 2.94]**	**2.2%**	—
**Random effects model**			**3.22**	**[1.83; 5.67]**	—	**23.4%**
Heterogeneity: $I^2 = 63\%$, $\tau^2 = 0.3011$, $p = 0.02$						
**Fixed effect model**			**2.29**	**[2.20; 2.38]**	**100.0%**	—
**Random effects model**			**2.25**	**[1.80; 2.82]**	—	**100.0%**

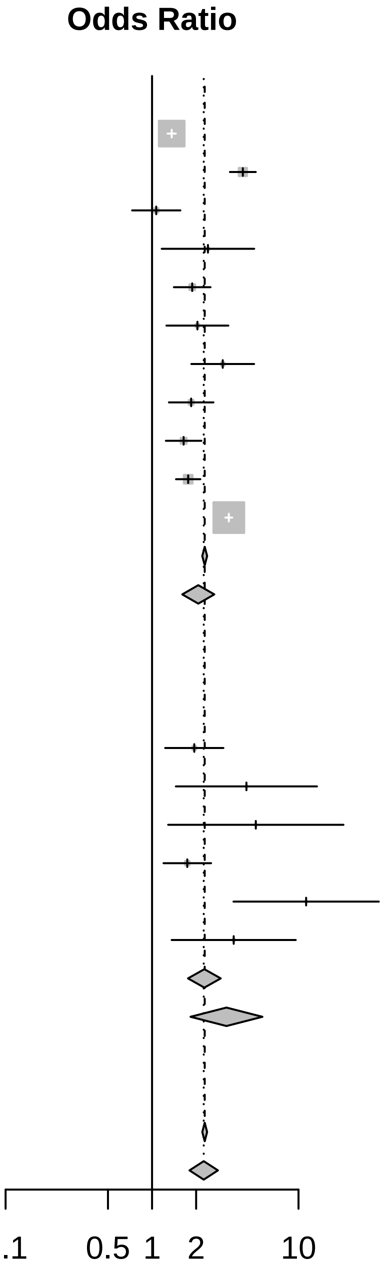

Heterogeneity: $I^2 = 97\%$, $\tau^2 = 0.1571$, $p < 0.01$

Test for subgroup differences (fixed effect): $\chi^2_1 = 0.00$, df = 1 ($p = 0.97$)

Test for subgroup differences (random effects): $\chi^2_1 = 1.97$, df = 1 ($p = 0.16$)

Supplementary figure 3. Subgroup analysis for malnutition-related risk of prevalent AKI by sample number
