# Supplementary material for: Association of Malnutrition with Risk of Acute Kidney Injury: A Systematic Review and Meta-Analysis
Source: Int J Clin Pract. 2023 Sep 26;2023:9910718. doi: 10.1155/2023/9910718 (PMC10547578; doi:10.1155/2023/9910718)
Supplement: Supplementary Materials — Supplementary Figure 1: subgroup analysis for malnutrition-related risk of prevalent acute kidney injury by region. Supplementary Figure 2: subgroup analysis for malnutrition-related risk of prevalent acute kidney injury by sample number. Supplementary Figure 3: subgroup analysis for malnutrition-related risk of prevalent acute kidney injury by age. Supplementary Figure 4: subgroup analysis for malnutrition-related risk of prevalent acute kidney injury by malnutrition assessment method. Supplementary Figure 5: subgroup analysis for malnutrition-related risk of prevalent acute kidney injury by patient characteristics. Supplementary Figure 6: subgroup analysis for malnutrition-related risk of prevalent acute kidney injury by covariate adjustment degree. Supplementary Figure 7: subgroup analysis for malnutrition-related risk of prevalent acute kidney injury by study quality. Supplementary Figure 8: sensitivity analysis for the risk of prevalent acute kidney injury in patients with malnutrition. Supplementary Figure 9: funnel plot for the risk of prevalent AKI in malnutrition patient. Supplementary Figure 10: sensitivity analysis for the risk of prevalent AKI in malnutrition patient. Supplementary Table 1: sensitivity analysis for the risk of prevalent AKI in malnutrition patient. [file 9910718.f1.zip › Supplementary Table 1. Sensitivity analysis for risk of prevalent AKI in malnutrition patient-R1 (1).docx]

**Supplementary Table 1. Sensitivity analysis for risk of prevalent AKI in malnutrition patient**

| **Omitting study** | **OR** | **95% CI** | **I^2^ (%)** | **P(z-text)** | **Effect model** |
| --- | --- | --- | --- | --- | --- |
| Khatana 2022 | 3.00 | (2.86-3.14) | 89 | < 0.01 | Random |
| Usta 2022 | 2.29 | (2.21-2.38) | 97 | < 0.01 | Random |
| Ying 2022 | 2.29 | (2.20-2.38) | 97 | < 0.01 | Random |
| Aykut 2022 | 2.29 | (2.20-2.38) | 97 | < 0.01 | Random |
| Wang 2022 | 2.24 | (2.15-2.33) | 97 | < 0.01 | Random |
| Li 2022 | 2.31 | (2.22-2.40) | 97 | < 0.01 | Random |
| Liang 2022 | 2.29 | (2.20-2.38) | 97 | < 0.01 | Random |
| Sertdemir 2021 | 2.29 | (2.21-2.38) | 97 | < 0.01 | Random |
| Kurtul 2021 | 2.28 | (2.20-2.37) | 97 | < 0.01 | Random |
| Dong 2021 | 2.30 | (2.21-2.39) | 97 | < 0.01 | Random |
| Chen 2022 | 2.29 | (2.20-2.38) | 97 | < 0.01 | Random |
| Efe 2021 | 2.29 | (2.20-2.38) | 97 | < 0.01 | Random |
| Wei 2021 | 2.28 | (2.20-2.37) | 97 | < 0.01 | Random |
| Han 2021 | 2.29 | (2.21-2.38) | 97 | < 0.01 | Random |
| Yu 2021 | 2.30 | (2.22-2.39) | 97 | < 0.01 | Random |
| Hu 2021 | 2.31 | (2.22-2.40) | 97 | < 0.01 | Random |
| Lu 2021 | 1.59 | (1.51-1.68) | 90 | < 0.01 | Random |

*Abbreviations:* OR, odd ratio; CI, confidence interval
