# Supplementary figures and images for: Association of Malnutrition with Risk of Acute Kidney Injury: A Systematic Review and Meta-Analysis
Source: Int J Clin Pract. 2023 Sep 26;2023:9910718. doi: 10.1155/2023/9910718 (PMC10547578; doi:10.1155/2023/9910718)

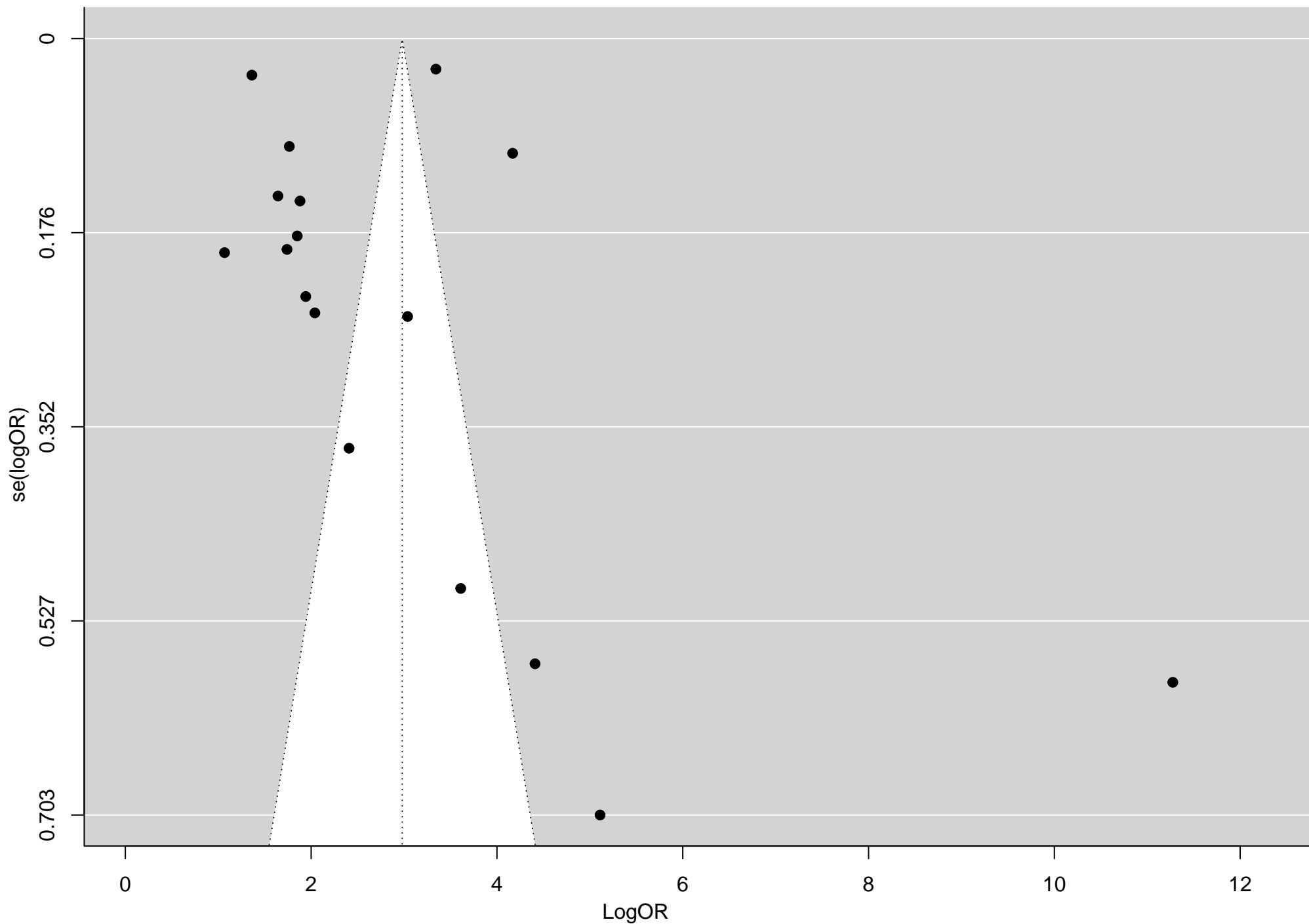

Supplementary figure 9. Funnel plot for risk of prevalent AKI in malnutrition patient

Supplement: Supplementary Materials — Supplementary Figure 1: subgroup analysis for malnutrition-related risk of prevalent acute kidney injury by region. Supplementary Figure 2: subgroup analysis for malnutrition-related risk of prevalent acute kidney injury by sample number. Supplementary Figure 3: subgroup analysis for malnutrition-related risk of prevalent acute kidney injury by age. Supplementary Figure 4: subgroup analysis for malnutrition-related risk of prevalent acute kidney injury by malnutrition assessment method. Supplementary Figure 5: subgroup analysis for malnutrition-related risk of prevalent acute kidney injury by patient characteristics. Supplementary Figure 6: subgroup analysis for malnutrition-related risk of prevalent acute kidney injury by covariate adjustment degree. Supplementary Figure 7: subgroup analysis for malnutrition-related risk of prevalent acute kidney injury by study quality. Supplementary Figure 8: sensitivity analysis for the risk of prevalent acute kidney injury in patients with malnutrition. Supplementary Figure 9: funnel plot for the risk of prevalent AKI in malnutrition patient. Supplementary Figure 10: sensitivity analysis for the risk of prevalent AKI in malnutrition patient. Supplementary Table 1: sensitivity analysis for the risk of prevalent AKI in malnutrition patient. [file 9910718.f1.zip › Supplementary figure 9. Funnel plot for risk of prevalent AKI in malnutrition patient (1).pdf]
